# Demystifying the Black Box: The Importance of Interpretability of Predictive Models in Neurocritical Care

**DOI:** 10.1007/s12028-022-01504-4

**Published:** 2022-05-06

**Authors:** Laura Moss, David Corsar, Martin Shaw, Ian Piper, Christopher Hawthorne

**Affiliations:** 1 Department of Clinical Physics & Bioengineering, NHS Greater Glasgow and Clyde, Room 2.41, Level 2, New Lister Building, Glasgow Royal Infirmary, 10-16 Alexandra Parade, Glasgow, G31 2ER UK; 2 School of Medicine, Dentistry and Nursing, University of Glasgow, Glasgow, UK; 3 School of Computing, Robert Gordon University, Aberdeen, UK; 4 Usher Institute of Informatics, University of Edinburgh, Edinburgh, UK; 5 Department of Neuroanaesthesia, Institute of Neurological Sciences, NHS Greater Glasgow and Clyde, Glasgow, UK

**Keywords:** Machine learning, Algorithms, Critical care, Artificial intelligence, Clinical decision-making

## Abstract

Neurocritical care patients are a complex patient population, and to aid clinical decision-making, many models and scoring systems have previously been developed. More recently, techniques from the field of machine learning have been applied to neurocritical care patient data to develop models with high levels of predictive accuracy. However, although these recent models appear clinically promising, their interpretability has often not been considered and they tend to be black box models, making it extremely difficult to understand how the model came to its conclusion. Interpretable machine learning methods have the potential to provide the means to overcome some of these issues but are largely unexplored within the neurocritical care domain. This article examines existing models used in neurocritical care from the perspective of interpretability. Further, the use of interpretable machine learning will be explored, in particular the potential benefits and drawbacks that the techniques may have when applied to neurocritical care data. Finding a solution to the lack of model explanation, transparency, and accountability is important because these issues have the potential to contribute to model trust and clinical acceptance, and, increasingly, regulation is stipulating a right to explanation for decisions made by models and algorithms. To ensure that the prospective gains from sophisticated predictive models to neurocritical care provision can be realized, it is imperative that interpretability of these models is fully considered.

## Introduction

A neurointensive care unit (NICU) is a cognitively challenging environment; large volumes of patient data are required to be analyzed, and decisions are rapidly made, often on the basis of uncertain information [1]. Monitoring systems found in the NICU capture streaming physiological data, which can be combined with electronic health record data, creating a data-rich environment. Data can be complex and heterogenous, structured and unstructured, and high and low frequency, and can also have quality issues, such as incompleteness and artifacts. Although analysis of these patient data has the potential to drive clinical knowledge discovery and aid in patient treatment, such a large volume of data is beyond the normal abilities of human cognition and hence cannot be analyzed easily.

To overcome this data challenge, techniques such as machine learning (ML) are increasingly applied and are becoming viewed as essential for supporting clinical decision-making [2]. ML is a subfield of artificial intelligence focused on the development of algorithms that extract patterns (or models) from large data sets, which can then be applied to other data for tasks such as prediction, prognosis, and classification [2]. The nature of NICU patient data means that ML is an ideal technique to be applied in this domain.

Although models derived from ML appear clinically promising, their interpretability has often not been considered. Applications in the NICU have focused on developing models that, when applied to new patient data, tell the clinician what is likely to happen for a particular scenario (e.g., mortality following traumatic brain injury [3]); the focus is on the *output* of the model rather than the *inner workings* of the model. The intrinsic interpretability of ML models range from techniques such as decision trees, which produce human-readable rules, to neural networks, which are often considered as black box approaches because they produce complex models that provide very little (or no) ability to comprehend the model created [4]. The model’s lack of interpretability and accountability is important, and alongside issues such as bias [5], lack of validation [6], ethics [7], and lack of technical infrastructure [8], it may contribute to reduced trust and clinical acceptance.

Recently, there has been a significant amount of societal interest in increasing the accountability of approaches such as ML; this has largely focused on opening-up black box algorithms to enable the user to understand the model. More widely, automated decision-making is increasingly being regulated, which has implications for health care, for example, the European Union directive General Data Protection Regulation stipulates the right to explanation for decisions made by algorithms, stating that individuals have the right to “meaningful information about the logic involved, as well as the significance and the envisaged consequences of such processing” [9].

At this point, it is worthwhile distinguishing between interpretation and explanation. A useful definition for these terms is provided by Miller [10]: interpretability can be considered as “how well a human could understand the decisions in the given context.” Applied to modeling, this can be thought of as how easy it is to identify cause and effect from the model’s inputs and outputs, i.e., *what* is happening in the model. For example, in the APACHE II model [11], a patient’s severity of disease is linearly calculated on the basis of the sum of points associated with 12 physiological variables taken on admission to critical care. The more points a patient has, the higher the patient’s disease severity. It is easy to see how the model uses the input data to make a final severity score. Models produced from ML approaches can represent complex relationships, but this complexity is also the reason they may not be interpretable. An explanation can be considered as answering questions such as ‘why’ and ‘how’ [10]. Explanation, within the context of ML, can be considered as a form of post-hoc interpretability [12]. To generate an explanation from a model, understanding and knowledge of the inner workings of the model is needed, i.e., it requires reasoning about the variables, which in turn requires domain knowledge and model context. With respect to the APACHE II model, you do not need to have intensive care unit domain knowledge to interpret how the score is calculated. However, to explain *why* (the higher the score, the more severely ill the patient is) requires knowledge and understanding of the model’s physiological variables and how the possible values of these variables relate to a patient’s condition.

The NICU can be considered as a high-risk application domain; patients are critically ill, and consequences of incorrect modeling are severe. By making the workings of models and algorithms explicit, it provides end users with the ability to understand and evaluate the model and interrogate it to detect possible issues, such as bias, incompleteness, and incorrectness. Further, removing some of the opaqueness associated with ML models can contribute to building clinicians’ trust and increase use of models in clinical practice [13].

## Neurointensive Care Models

A wide variety of models are relevant to the NICU and range in interpretability. The majority of those in clinical use are not yet from black box ML algorithms but from what is thought of as classical statistics. It is still worth exploring these from the perspective of interpretability to enable comparison. Further, it is acknowledged that there is no hard boundary between statistical inference and ML, and some methods fall into both domains [14, 15].

Classification models are often easily interpretable. The Glasgow Coma Scale Score [16] combines the findings of the three components of the Glasgow Coma Scale into a single index that can classify the patient’s condition as mild, moderate, or severe for the purposes of research studies. How the final score is calculated is immediately transparent to the user.

Prognostic models relate a patient’s characteristics with their risk of a particular future outcome. The International Mission for Prognosis and Analysis of Clinical Trials in Traumatic Brain Injury [17, 18] and the Corticosteroid Randomisation After Significant Head Injury [19] models are based on logistic regression analysis, and the sum scores and calculation of the probability of 6-month outcome are available for scrutiny.

Cerebral autoregulation (AR) can be modeled using physiological data in correlation methodologies and mathematical models [20]. The pressure reactivity index (PRx) and low-frequency autoregulation index (LAx) are relatively intuitive AR models. PRx is calculated on high-frequency data (LAx on minute-by-minute data) as a moving Pearson correlation between intracranial pressure and arterial blood pressure and assigns a value between − 1 and 1; 0 implies no correlation; 1 is positive correlation, in which pressure reactivity is impaired; and − 1 is negative correlation, in which pressure reactivity is intact [21]. Specialist software enables the values and correlation to be visualized [22]. Optimum cerebral perfusion pressure (CPPopt) for an individual patient is calculated by combining continuous monitoring of cerebral perfusion pressure and a measure of AR through a process of data thinning and collation, resulting in a quadratic polynomial linear regression model fit [23, 24]. Although the software can display an almost continuous estimation of CPPopt, the underlying model requires more advanced knowledge to interpret.

Models are also used for analysis of electroencephalogram signals for monitoring depth of anesthesia and sedation [25, 26]. The Bispectral Index (BIS) monitor (Medtronic) collects raw electroencephalogram data, and a multivariate model using nonlinear functions of electroencephalography-based subparameters calculates the BIS score (i.e., the prediction of depth of anesthesia). The BIS is an example of another type of model opaqueness; in this case, the interpretability of the model is poor owing to only the principles and not the specifics of the proprietary algorithm being publicly available.

More recently, techniques from the field of ML have been applied to NICU data to create predictive models (e.g., prediction of neurological recovery). For a more detailed overview of ML in the NICU see the article by Chaudhry et al. [14]. Models derived from ML are often more complex, are much less intuitive, and are not yet widely applied in the NICU. For example, a neural network can consist of multiple layers of artificial neurons with possibly thousands of parameters. An ML expert could conceptually explain what is happening in the layers, but it is effectively impossible to follow the computation and explain how all the thousands of parameters worked together to generate the prediction. As ML models become more prevalent in NICU clinical practice, this lack of interpretation is problematic.

## Interpretable ML

To overcome the black box nature of some ML models, there is a growing interest in the use of interpretable ML. There are generally three different approaches to developing interpretable models [27]: firstly, to use models that are intrinsically understandable by a human; secondly, to apply interpretation methods after model creation using model-agnostic methods; and thirdly, for example-based methods to use an instance from the data to explain the behavior of the model. For a more detailed overview of interpretable ML, see the articles by Molnar [27] and Linardatos et al. [28], and for an overview of its benefits for health care, see the article by Ahmad et al. [29].

Using intrinsically understandable models offers the easiest approach to model interpretability. One such model is a decision tree; each node of the tree contains a question, and every child node (or “branch”) contains a possible answer to the question. To interpret the model, starting with the root node, the branches are followed through the child nodes until reaching the predicted outcome on the leaf node. Each node is a subset of the data, and each edge is an AND function. The advantage is that a tree structure is an intuitive visualization and lends itself to human-friendly interpretations [30]. Disadvantages include an inability to represent linear relationships and that the tree can quickly become too large. More generally, a disadvantage is that intrinsically understandable models may not offer the same accuracy as nonintrinsically understandable models.

Using post-hoc model-agnostic methods means that a modeler does not have to restrict themselves to intrinsically interpretable ML algorithms nor to one type of interpretation. Post-hoc interpretation methods may explain an individual patient prediction (local interpretation) or the whole model (global interpretation).

Local interpretable model-agnostic explanations (LIME) [31] is a model simplification technique, creating a local interpretable surrogate model to represent what is happening within a black box algorithm. This is done by changing the black box algorithm inputs and creating a new data set consisting of the perturbed samples and predictions. Using this data set, LIME trains an interpretable model weighted by the closeness of the new instances to the original instances. LIME has the advantages that it generates human-friendly explanations; works for tabular, text, and image data; and is relatively easy to use, whereas disadvantages include manual experimentation with different kernel settings for each application and a lack of stability in the explanations generated [28].

Other post-hoc model-agnostic methods focus on feature importance. Shapley Additive Explanations (SHAP) calculates an additive feature importance score for each model prediction [32]. Shapley values is an approach from cooperative game theory that fairly attributes how much each of a model’s input features contributed to its output. SHAP values refer to Shapley values that have been applied to a conditional expectation function of an ML model. An advantage to this approach is that it is built on solid theory and has desirable properties (local accuracy, missingness, and consistency). Disadvantages include requiring a large amount of computing time, returning only a value rather than a model (which limits answering ‘what-if’ questions), and generating explanations using all the features rather than sparse explanations [28].

Example-based methods are generally model-agnostic methods and help a human to create a mental model of the ML model and the data it has been trained on [28]. Using examples to reason with is common in everyday life; we often use what we know from similar situations to make inferences about a current situation. For example, counterfactual explanations of a prediction identify the feature value changes required to change the output of the model [33]. Example-based methods work well if a feature value is associated with context and represented in a human understandable way (e.g., text), whereas they can be harder to apply to tabular data because an instance can consist of many features that are unstructured [28].

Model interpretations should be helpful to a human achieving a given objective. Doshi-Velez and Kim [34] suggest a taxonomy of evaluation approaches: application grounded (involving domain expert experiments to evaluate the quality of the interpretation within the context of its end-task, e.g., identification of errors), human grounded (involving simpler human-subject experiments in which more general notions of a good interpretation are evaluated, e.g., which interpretation is preferred under time constraints), and functionally grounded (involving no humans and instead focusing on a formally defined notion of interpretability as a proxy, e.g., showing that your model performs better with respect to sparsity).

Although not widely applied, interpretable ML algorithms have been applied to critical care data, largely focusing on prediction of patient outcome. Table 1 summarizes some of these applications.

**Table 1:** Selected examples of the use of interpretable machine learning approaches to NICU data

Article	Study population	Data set(s)	Predicted variable	Machine learning algorithm applied	Interpretability technique(s)
Overweg et al. [40]	ICU and TBI	CENTER-TBI, MIMIC-III	ICU/NICU mortality	BNN	HorseshoeBNN—a novel approach proposed by the authors; the horseshoe prior has been added to induce sparsity in the first layer of the BNN, enabling feature selection
Caicedo-Torres and Gutierrez [41]	ICU	MIMIC-III	ICU mortality	Multiscale deep convolutional neural network (ConvNet)	DeepLIFT, visualizations
Thorsen-Meyer et al. [42]	ICU	5 Danish medical and surgical ICUs	All-cause 90-day mortality	Recurrent neural network with LSTM architecture	SHAP
Wang et al. [43]	ICU patients diagnosed with cardiovascular disease	MIMIC-III	Survival	LSTM network	Counterfactual explanations
Fong et al. [44]	ICU	eICU collaborative research database and 5 ICUs in Hong Kong	Hospital mortality	XGBoost	SHAP
Che et al. [45]	Pediatric ICU patients with acute lung injury	Pediatric ICU at Children’s Hospital Los Angeles	Mortality, ventilator-free days	Interpretable mimic learning (using gradient boosting trees)	Partial dependence plots, feature importance, intrinsic interpretability of tree structure
Shickel et al. [46]	ICU	UFHealth, MIMIC-III	In-hospital mortality	RNN with GRU	Modified GRU-RNN network with final self-attention mechanism (to identify feature importance)
Farzanah et al. [47]	TBI	ProTECT III	Functional outcome – GOSE at 6 months	XGBoost	SHAP
Gao et al. [48]	TBI	NICU at Cambridge University Hospitals, Cambridge	Mortality 6 months post brain injury	Decision tree	Intrinsic interpretability of model
Thoral et al. [49]	ICU	AmsterdamUMCdb	ICU readmission and/or death, both within 7 days of ICU discharge	XGBoost	SHAP

## Discussion

Models and algorithms can significantly advance NICU patient treatment. As the NICU becomes more technology driven and growing amounts of patient data become available, ML provides a valuable tool with which to analyze these data, but models need to be accessible and accountable to aid adoption into NICU clinical practice.

Interpretable ML is an approach that has the potential to enhance the utility and acceptance of ML models, but there are challenges. Compared with black box ML approaches, there can sometimes be a reduction in performance when applying intrinsically interpretable ML models. Consequently, for a given task, there can be a trade-off between accuracy and the intrinsic interpretability of the model. For example, in the article by Cowan et al. [35], a decision tree model to predict intensive care unit patient hospital survival led to poorer discriminative ability than several black box algorithms, and in the article by Holmgren et al. [36], an artificial neural network outperformed the Simplified Acute Physiology Score 3, which is built using linear regression. However, a reduction in model performance when using interpretable ML is not always found [37].

The requirements for interpretability of ML models and the acceptable trade-off between model accuracy and interpretability may depend on the context in which the model is being applied. For example, a model to predict whether a patient is likely to survive the NICU has the potential to substantially influence the treatment of a patient; no matter how accurate the model, it is unlikely that a clinician will fully trust the model without requiring understanding of how the model came to its conclusion. However, there will be more subtle scenarios for which the trade-off between accuracy and interpretability is not as straightforward. Little research has been conducted to examine such scenarios and to explore, within the NICU context, what are acceptable trade-offs. It is proposed that this will be context specific and may differ between clinicians on the basis of attitude to risk and experience level. How a model is being used by clinicians may also be important, for example, if a clinician is using the output of a model with other sources of information, then the requirement for a more accurate model may be stronger than the requirement for a more interpretable one because the clinician will not be relying solely on the model for the decision-making.

Interpretability can be applied to multiple stages of the modeling process to provide transparency of the algorithm’s process and aid comprehension of the model or understanding of how parts of the global model affect predictions, understanding of a single model prediction, and interpretation of model predictions for multiple instances [28]. The way that interpretability is presented to the end user is highly dependent on the chosen algorithm. There is no agreed-on definition of interpretability, nor is there agreement on which characteristics define a useful interpretation for NICU clinicians. Previous research has focused on developing the techniques to provide interpretation mechanisms rather than identifying what would be considered a useful or valid interpretation of a model [34]. Consequently, there can be a disconnect between a clinician’s requirements for interpreting an ML model and existing notions of interpretable ML [38]. Further, interpretable ML techniques indicate the parts of the data that contributed most to an ML model’s output but cannot indicate why (i.e., offer an explanation). It is important to be aware of this limitation because the clinician is left to make their own interpretation of what they think the model reasoning was, and this may be biased by their own prior knowledge and experience [39]. It is suggested that an international consensus is developed regarding the requirements for interpretation of predictive models in the NICU and for the standardization of approaches.

Finally, some post-hoc interpretable ML approaches (e.g., LIME) are often more time and resource intensive, compared with intrinsically interpretable ML approaches, and often require expertise to implement; if a computer system applies a model and then subsequently generates an explanation each time it is used, this may reduce the overall system performance. If interpretable ML approaches are to be adopted in clinical practice, they will need to overcome real-world NICU requirements and run in clinically useful timescales despite limited computing infrastructure and processing power.

It is important from the perspective of patient safety, ethics, and accountability that algorithms that can be used to influence patient treatment are “opened up” and that attempts are made by those who use them to understand the underlying processes. Interpretable ML methods provide one solution to this problem, but there is also an onus on NICU education and training to keep up to date with analytical and technological advances so that clinicians can understand not only the benefits of these approaches but also the limitations. Blind faith in black box models is something that the NICU community needs to be aware of and should be seeking to avoid.
